# Dynamic Control of Nucleic Acids Self‐Assembly and Expression Using Photoswitches

**DOI:** 10.1002/chem.71079

**Published:** 2026-05-06

**Authors:** Noemí Nogal, Hugo Laigle, Maxime Mansy, Sébastien Ulrich, Mathieu Surin

**Affiliations:** ^1^ Department of Chemistry and Biomolecular Sciences University of Ottawa Ottawa Ontario Canada; ^2^ Institut des Biomolécules Max Mousseron (IBMM), CNRS, ENSCM Université de Montpellier Montpellier France; ^3^ Laboratory For Chemistry of Novel Materials Center of Innovation and Research in Materials and Polymers (CIRMAP) University of Mons Mons Belgium

**Keywords:** nucleic acids, photocontrol, photoswitches, self‐assembly, supramolecular chemistry

## Abstract

Synthetic nucleic acids have become readily available and now constitute versatile building blocks in materials science—where they can be used to engineer DNA origami—and biotechnologies, where they act as bioactive entities. It has therefore become of interest to develop chemical approaches enabling an external control over their function. In this line, photoswitches have attracted a strong interest since light is a benign stimulus which can be applied with spatio‐temporal precision. In this review, we showcase the recent evolution in the design of photoswitches with improved performances, and highlight selected applications of their combination with nucleic acids.

## Graphical abstract

We review here the recent progress made in the design of molecular photoswitches, and highlight their implementation for the dynamic control over nucleic acids self‐assembly and expression.

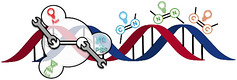

## 1 Introduction

The ability to control the structures of biomolecules and their interactions in a reversible manner to modulate the complex processes that take place in live cells is a major scientific goal at the crossroad of chemical biology, biophysics, and biomedicine [1, 2, 3].

As an external stimulus, light is an easy‐to‐use trigger for controlling the configuration of specific photo‐switchable molecules, which can interact with various biochemical systems noninvasively (in a vast range of wavelengths) in a reversible manner [1]. Importantly, the wavelength and light intensity required for performing the photoswitching can be precisely tuned by molecular design in order to reach the therapeutic window [4]. When considering interfacing photoswitches with nucleic acids, one should consider discarding the use of high ‐energy UV light, which can photodegrade and create cross‐links in nucleic acids. Besides, the light intensity used to activate the photoswitches and the duration of light exposure can be precisely adjusted, thus allowing control of the degree of activation of the photoswitches and thus the gradual or partial activation of the photosensitive systems at a specific location (spatial control) [5].

Photoswitches have garnered significant interest for bioapplications due to their unique ability to reversibly modulate the structural and chemical properties in response to light stimulation [6, 7, 8]. This offers several advantages for biological and medical applications, such as the time‐ and space‐defined regulation of biological processes like enzyme activity, ion channel function, and gene expression; controlling drug activity in photopharmacology; modulating material properties in smart materials and nanostructures, and developing light‐controlled catalysts and molecular machines.

In this context, researchers have introduced photoswitches into bioactive compounds [9], including synthetic nucleic acids and nucleic acid binding ligands. In this mini‐review, we highlight the recent advances made using photoswitches for gaining control over the structure and function of nucleic acids. First, we describe common and recently developed photoswitches used to modulate biomolecular structures with light, in particular for their use with nucleic acids. Then, we discuss the covalent modification of nucleic acids with photoswitches utilized e.g., to control the DNA‐DNA hybridization processes with light. We also report on the design of photoswitches that bind to nucleic acids through supramolecular interactions, and their utilization to play with the dynamics of self‐assembly, toward higher‐order DNA structures. Finally, we conclude and offer some perspectives on possible improvements and applications at the interface of biology with chemistry, toward health sciences.

## 2 Molecular Photoswitches for Biological Applications

**FIGURE 1:**
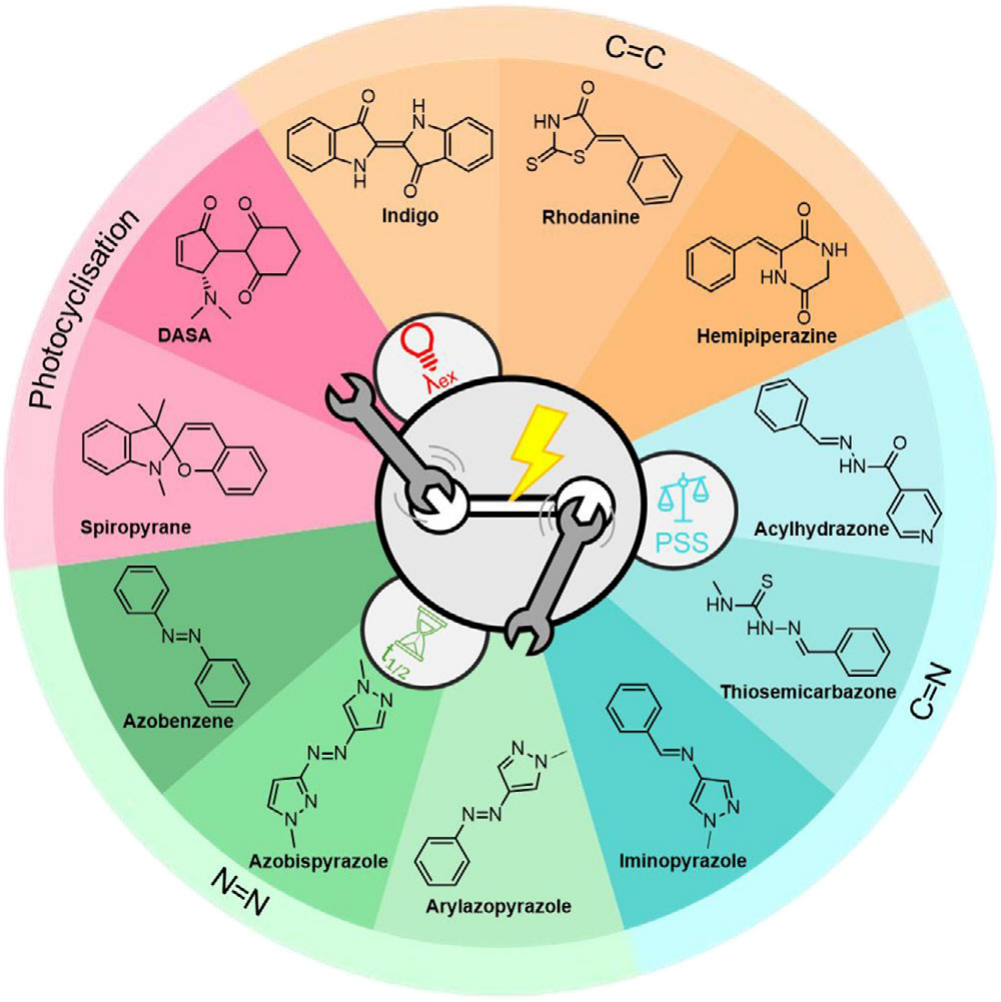
Graphical representation of molecular photoswitches discussed in this review. Critical features include *λ*_e__x_ corresponding to the excitation wavelength, PSS corresponding to the photostationary state composition, and t_½_ corresponding to the thermal half‐life.

A variety of synthetic photoswitches that can undergo reversible conformational change upon irradiation have been designed. In this section, we present a selection of photoswitchable groups with interesting optical properties in terms of excitation wavelength, photo‐stationary state (PSS), and half‐life (t½), making them of interest for interactions with nucleic acids (Figure 1). The discussion highlights the structural effect which enable tuning their optical and photophysical properties.

### 2.1 Common Photoswitches

**FIGURE 2:**
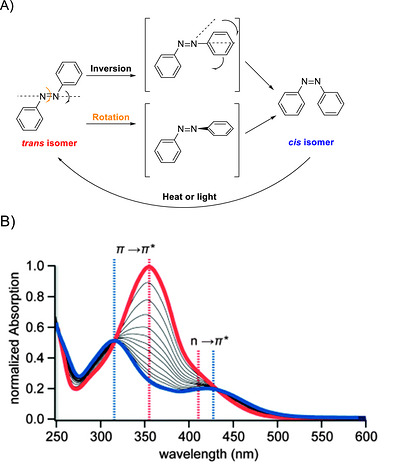
(A) Photoisomerization mechanisms of azobenzenes (B) UV‐Vis absorption spectra of the *cis*‐ and *trans*‐isomers with their corresponding electronic transitions. Figure adapted from reference [11].

In this first part, we present relevant aspects of the isomerization processes of the most popular photoswitches, namely azobenzenes and spiropyrans. The photoisomerization process of azobenzenes from the stable *trans* (*E*) isomer can be interconverted to the *cis* (*Z*) isomer by irradiation with UV light involves the rotation around the N = N double bond (Figure 2A) [10]. The nonbonding electron pair of each nitrogen atom can give rise to an electronic transition n‐π*(S_0_→S_1_) with inversion of the nitrogen atom. The isomerization can also occur via a rotation mechanism, in which a π‐π* (S_0_→S_2_) transition occurs, Figure 2 (panel A).

The UV‐Vis spectra of azobenzenes show two characteristic bands, corresponding to the n‐π* and π‐π* electronic transitions. The *trans* isomer has a very intense π‐π* absorption band, with a molar extinction coefficient (ε) around 3 × 10^4^ M^−1^ cm^−1^, while the second band (n‐π*) appears much weaker, on the order of ε ∼ 400 M^−1^ cm^−1^. In contrast, the corresponding *cis* isomer has a π‐π* band shifted to shorter wavelengths (hypsochromic effect), considerably decreasing in intensity (ε ∼ 10 × 10^3^ M^−1^ cm^−1^); in turn, the n‐π* electronic transition (380‐520 nm) in the *cis* isomer is allowed, which results in an increase of its intensity (ε ∼ 1500 M^−1^ cm^−1^) with respect to the *trans* isomer (Figure 2B).

The precise measures of these properties are important for translating photoswitching events into biological effects. In such a context, recent photoswitches are developed to absorb in the near‐infrared range (700–900 nm) or in the visible region (400–700 nm), that is, wavelength ranges which can effectively penetrate biological tissues. These ‘red‐shifted’ systems allow incident light to cause conformational changes in the molecule without interfering with surrounding biological components. The PSS is a crucial aspect for biological applications, as the aim is for the system to reach a steady state with the largest possible amplitude at the required wavelength. The kinetics of photoswitching is another critical aspect, which is affected by the light intensity as well as the nature of the aqueous medium and its crowding effect. In general, the photoswitching process is intended to occur on time scales that are relevant to biomolecular dynamics, typically in the range of milliseconds to seconds [9]. The thermal stability of photoswitches shall not be neglected, as it must be ensured that the system remains inactive and does not degrade in physiological temperatures before its activation by light. Thus, a photoswitch must be thermostable at body temperature (37 °C). Therefore, all these thermodynamic and photophysic features should be carefully considered when selecting photoswitches for biological applications.

Recently, azobenzene units were incorporated into oligonucleotides, enabling light‐induced modulation of DNA triplex formation and thereby allowing photochemical control over nucleic‐acid architecture (vide infra). [12]

**FIGURE 3:**
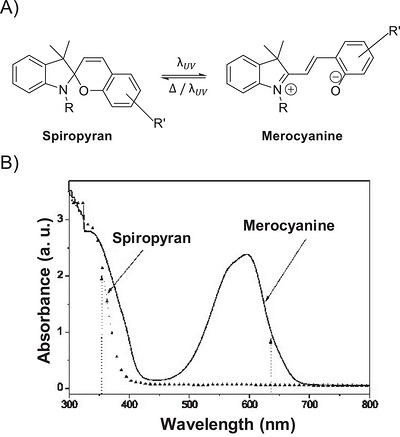
(A) Structures of spiropyran and merocyanine. (B) UV‐Vis absorption spectra of the spiropyran and merocyanine forms. Figure adapted from reference [16].

Some photoswitchable compounds owe their activity to electrocyclisation processes, as for instance spiropyrans that absorb in the visible range (see example Figure 3). An electrocyclic reaction is a type of reversible intramolecular cycloaddition in which the formation of σ bonds occurs from conjugated π electrons in a neutral or ionic system [13] by a concerted mechanism. These reactions require a triggering by temperature or radiation for interactions to occur between the p orbitals that form the double bonds between the chemical systems involved, according to the Woodward‐Hoffmann rules [14]. Electrocyclic reactions allow stereospecificity to be induced by using light or heat.

Commonly called BIPS (1’,3’‐dihydro‐1’,3’,3’‐trimethyl‐spiro[2H‐1‐benzopyran‐2,2’‐(2H)‐indole]), their structure consists of two heterocyclic parts linked by a tetrahedral sp3 carbon atom and a 2‐methylene‐1,3,3‐trimethylindoline derivative, Figure 3 (panel A). The absorption spectra of these molecules show a signal in the UV range, with a maximum absorption band between 320–380 nm, Figure 3 (panel B). When the C‐O bond of the spiropyran is broken during the isomerization process, it gives rise to the “open” or “colored” form of the active compound, also called merocyanin, which absorbs in the visible region in the range of 500–600 nm, Figure 3B. From its open form (merocyanin), reversion to its original or closed form can occur thermally or photochemically, by applying visible or UV radiation. In the excited state, it can undergo secondary photodegradation reactions more easily. In addition, atmospheric oxygen facilitates these degradation processes by forming free radicals that interact with the spiropyran molecule. The merocyanine form, in a biological context, switch has been found to interact strongly with model proteins [15].

**FIGURE 4:**
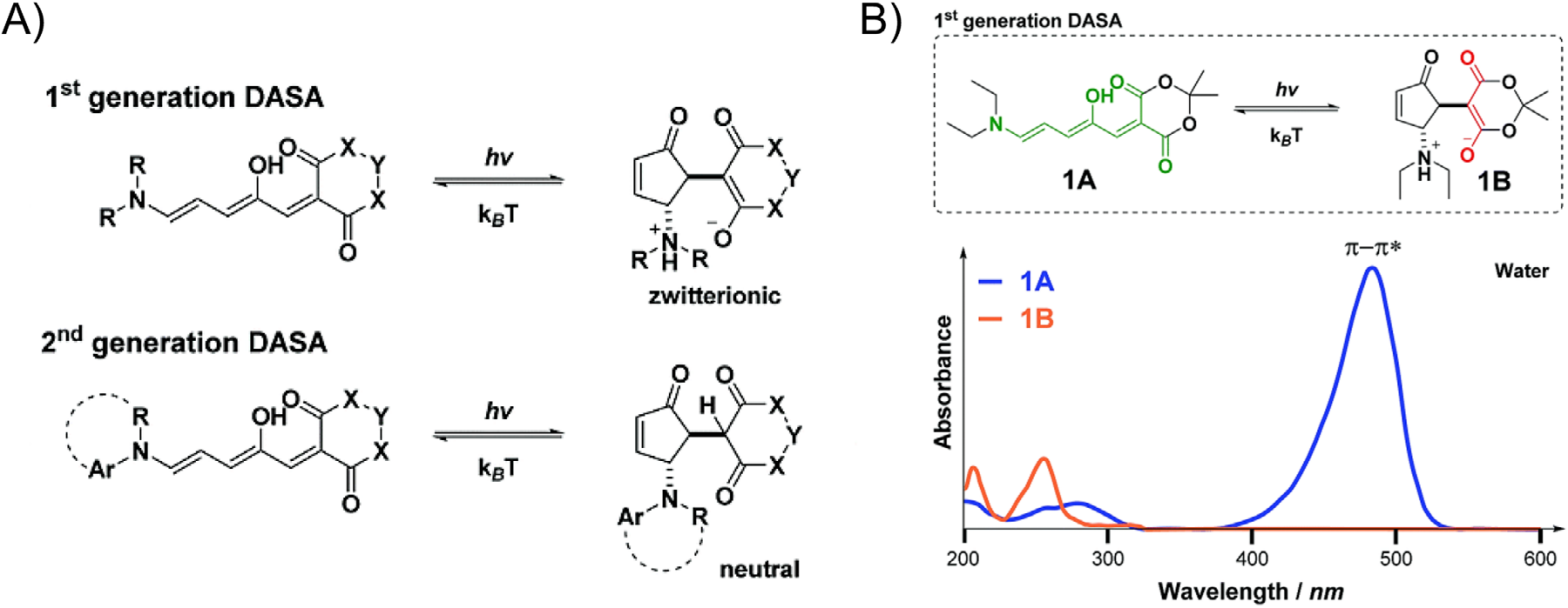
(A) Structure and photoswitching process of a donor‐acceptor Stenhouse adducts (B) Schematic representation of the absorption spectrum of compound 1 for both the elongated triene form A and the cyclopentenone form B in water. The push–pull system (green in 1A) and the 1,3‐dicarbonyl system (red in 1B), are highlighted. Figure adapted from reference [17].

Other promising photoswitching compounds are donor‐acceptor Stenhouse adducts (DASAs). DASAs were first reported in 2014 by Alaniz and co‐workers, and were improved two years later with a second generation (Figure 4A) [17]. Structurally, the donor motif connects to the acceptor through a triene bond, which makes DASA a push‐pull system. This molecular build‐up is a key feature governing most of DASA photochemistry and helping their synthesis. The photoconversion of this system is governed by the photoisomerization of the C2‐C3 carbons adjacent to the hydroxyl group and the subsequent rotation around the C3‐C4 bond. it's the UV‐Vis spectrum shows a large absorption band around 500 nm due to the π‐π* transition, Figure 4B) this wavelength range being of interest for applications in biological science.

### 2.2 Recently‐Developed Photoswitches

Promising systems involving a photoinduced isomerization around N = N, C = C, and C = N bonds have emerged during the last decades, and their structure‐optical property relationships have been progressively studied and optimized to develop efficient photoswitches. Although biological applications remain hitherto scarce, they may be considered for the interactions with nucleic acids.

#### 2.2.1 N = N Photoswitches

**FIGURE 5:**
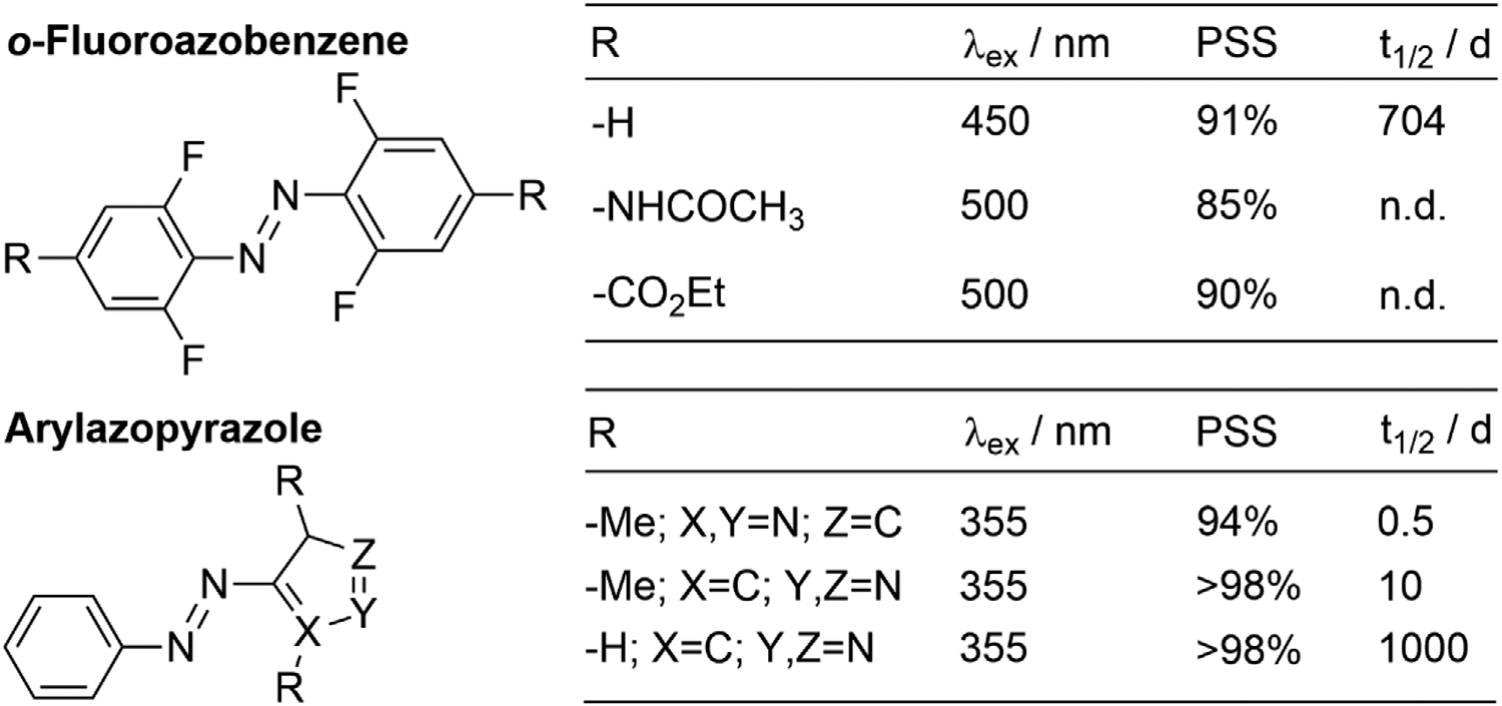
Molecular structures and optical properties of photoswitches involving N = N bond isomerization. λ_e__x_ corresponds to the excitation wavelength applied to the most thermodynamically stable isomer; PSS is the photostationary state composition after irradiation of this isomer; t_1/2_ represents the thermal half‐life of the less stable isomer.

***o*‐Fluoroazobenzene**. As described above, azobenzenes are one of the most used organic photoswitches due to their robust photochemical stability and facile synthesis. However, their limitations for biological applications come from the need for UV light irradiation and poor *E*/*Z* band separation, resulting in limited reversible *Z*→*E* photoisomerization (i.e., low PSS) due to the overlapping absorption bands of the n→π* transition between isomers. To overcome this issue, azobenzenes have been decorated with functional groups such as amines [18], methoxy groups [19], and fluorine atoms (Figure 5) [20]. Among these modifications, introducing fluorine groups offers the advantage of preserving the molecule planarity and π‐conjugation while significantly enhancing n→π* band separation. This improvement enables efficient photoswitching in the visible range (>500 nm) and in both directions [21]. Further structural modifications of *o*‐fluoroazobenzenes in *para* positions must be carefully designed, as in some cases the N = N bond can be sensitive to thiols and glutathione reduction depending on the pH [22]. However, additional changes also allow for near quantitative PSS, customizable half‐lives [23], and even enhanced water solubility, making them suitable for biological applications [24, 25, 26].

**Arylazopyrazole**. The development of arylazopyrazoles (Figure 5) as molecular photoswitches follows the same rational structural variations as iminopyrazoles. *Ortho*‐pyrrolidine and piperidine decorated phenyl rings enable red shift in both directions, while *N*‐methylation of the pyrazole enhances the thermal stability of *Z* isomers [26]. Moreover, altering the position of nitrogen atoms within the heterocycles and transitioning to azobispyrazoles both allow the tuning of π conjugation. These modifications enable control over half‐lives ranging from seconds to years, while also achieving near‐quantitative PSS values in solution [27, 28], in solids [29], and in crystals [30]. Structural design enables water solubility of arylazopyrazoles, whose N = N bond can be protonated to control pH‐dependent *Z* → *E* thermal or photoinduced switch [31, 32]. Recently, arylazopyrazoles were functionalized with nucleobases, enabling their photomodulated assembly along DNA templates (vide infra) [33].

We agree that redox stability is an important consideration for azo‑based photoswitches designed for biological applications. In classical azobenzenes, substitution patterns such as *ortho*‑fluoro significantly enhance photochemical addressability and can influence susceptibility to reductive cleavage; for example, certain *ortho*‑substituted azobenzenes that absorb visible light also show improved resistance to glutathione‑mediated reduction compared to electron‑rich analogues prone to rapid GSH attack [27, 34]. In contrast, arylazopyrazoles, have been shown to exhibit both quantitative photoisomerization and exceptionally long *Z*‑isomer thermal lifetimes, and studies have indicated that incorporation of heteroaryl rings can also enhance resistance to reductive degradation, such as that mediated by intracellular thiols like GSH under biologically relevant conditions [35, 36]. Finally, when using photo‐switches in aqueous media, careful consideration of pH effects should also be taken into consideration since the kinetics of isomerization of some azo‐based photoswitches can be greatly impacted [37].

#### 2.2.2 C = C Photoswitches

**FIGURE 6:**
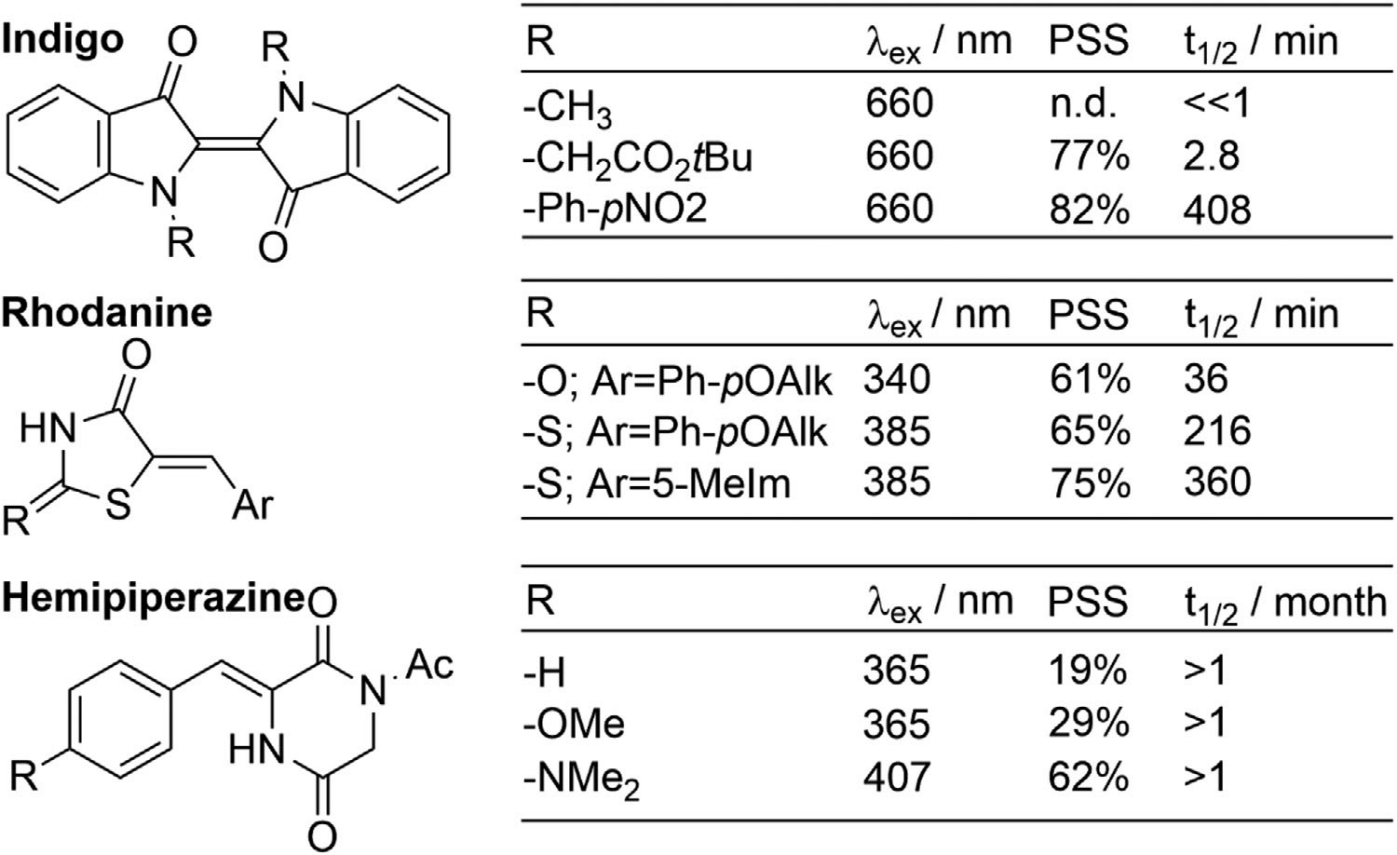
Molecular structures and optical properties of photoswitches involving C = C bond isomerization. λ_e__x_ corresponds to the excitation wavelength applied to the most thermodynamically stable isomer; PSS is the photostationary state composition after irradiation of this isomer; t_1/2_ represents the thermal half‐life of the less stable isomer. Ph stands for phenyl ring, p for para position, Alk for alkyl chain, and Im for Imidazole.

**Indigo**. Indigo structures (Figure 6), known for decades for their ability to undergo photoswitching under red light (660 nm), have been significantly optimized. By incorporating electron‐withdrawing *N*‐aryl groups, their half‐life (t_½_) has been extended from less than 5 s to up to 400 h without altering their absorption spectrum. These structures are easily functionalized from very cheap dyes, thus granting access to a wide range of compounds with absorption properties within the therapeutic light absorption window (600–1200 nm) [38]. Additional chemical modifications of these structures, such as *N*‐alkylation of the pyrrolidinone, can induce even more red‐shifted photoswitch in both *E*→*Z* and *Z*→*E* directions [39, 40]. Hemiindigos also bring improvements in photophysical properties by breaking the symmetry of indigos with other decorated aromatic cycles. These structural changes extend the π‐conjugation system and modify the molecular planarity, leading to improved absorption band separation, fatigue resistance, and tunable thermal stability of metastable states, ranging from picoseconds to years [41, 42, 43, 44]. Further structural modifications of hemiindigos allow solubility in aqueous media while retaining photoswitching capacity, with additional pH‐responsive switching, which is essential when considering potential biological applications [45, 46].

**Rhodanine**. Rhodanine‐based structures (Figure 6) exhibit excellent half‐lives, which can be tuned from 30 min to several days, for instance, by substituting oxygen with sulfur. This effect is attributed to differences in electronegativity and polarizability between the heteroatoms, which alter their hydrogen‐bonding capacity and induce red shifts up to 57 nm in the absorption spectrum. The incorporation of substituted aromatic rings further modulates thermal stability and enables nearly quantitative photo‐stationary states (PSS). Although their activation primarily occurs in the UV‐Vis region near 400 nm, some derivatives display good cellular penetration and can induce apoptosis in a configuration‐specific manner [47].

**Hemipiperazine**. The hemipiperazines (HPIs, Figure 6) reported by Pianowski's group are worth mentioning [48, 49]. Modulations of the 3‐arylidene‐2,5‐diketopiperazine core with electron‐rich substituents enabled improvements in photoconversion and red‐shifting of the *λ*_max_ by up to 135 nm that made them promising as reversible fluorescent probes in cells with thermal‐isomerization mediated by glutathione [50], or for the treatment of solid tumors. For instance, plinabulin derivatives exhibit photomodulable reversible biological activity, with up to 1,800‐fold difference in activity between *E* and *Z* configurations, and high half‐lives of the less active E form for up to one month, highlighting their potential as pro‐drugs [48, 51].

#### 2.2.3 C = N Photoswitches

**Hydrazone and Acylhydrazone**. Aprahamian's group has described hydrazones as an excellent toolbox for photoswitching [52]. Intramolecular H‐bonds with the hydrazone amide hydrogen stabilize the *Z* isomer, enabling half‐lives up to thousands of years with quantitative PSS and good absorbance band separation in various forms: solution [53], solid‐state [54], liquid crystals [55], and nanoparticles with drug release [56]. Acylhydrazones, pioneered by the group of Hecht [57], exhibit optical properties similar to hydrazones while achieving, for the best ones, band separation of over 100 nm with quantitative PSS in both directions and long half‐lives without requiring *Z*‐isomer stabilization (Figure 6) [58]. Moreover, extension of the π‐conjugation with larger aromatic cycles, from phenyl to naphthyl, anthryl, and pyrenyl, leads to red shifts of the absorption bands up to 100 nm [57]. Finally, acylhydrazone photoswitching properties can be controlled through redox—the presence of free thiols catalyzing the isomerization via reversible addition onto the C = N bond—or pH modulations of ionizable ortho substituents [59, 60, 61]. These optical properties make carefully‐designed acylhydrazones promising photoswitches for biological applications, provided *E* and *Z* configurations display different biological effects. Examples were already described as photoswitchable ion pair transporters [62, 63]. Recently, hydrazone‐modified cytidine was developed as a photoswitchable nucleobase, enabling blue‐light control of DNA synthesis and polymerase activity [64].

**FIGURE 7:**
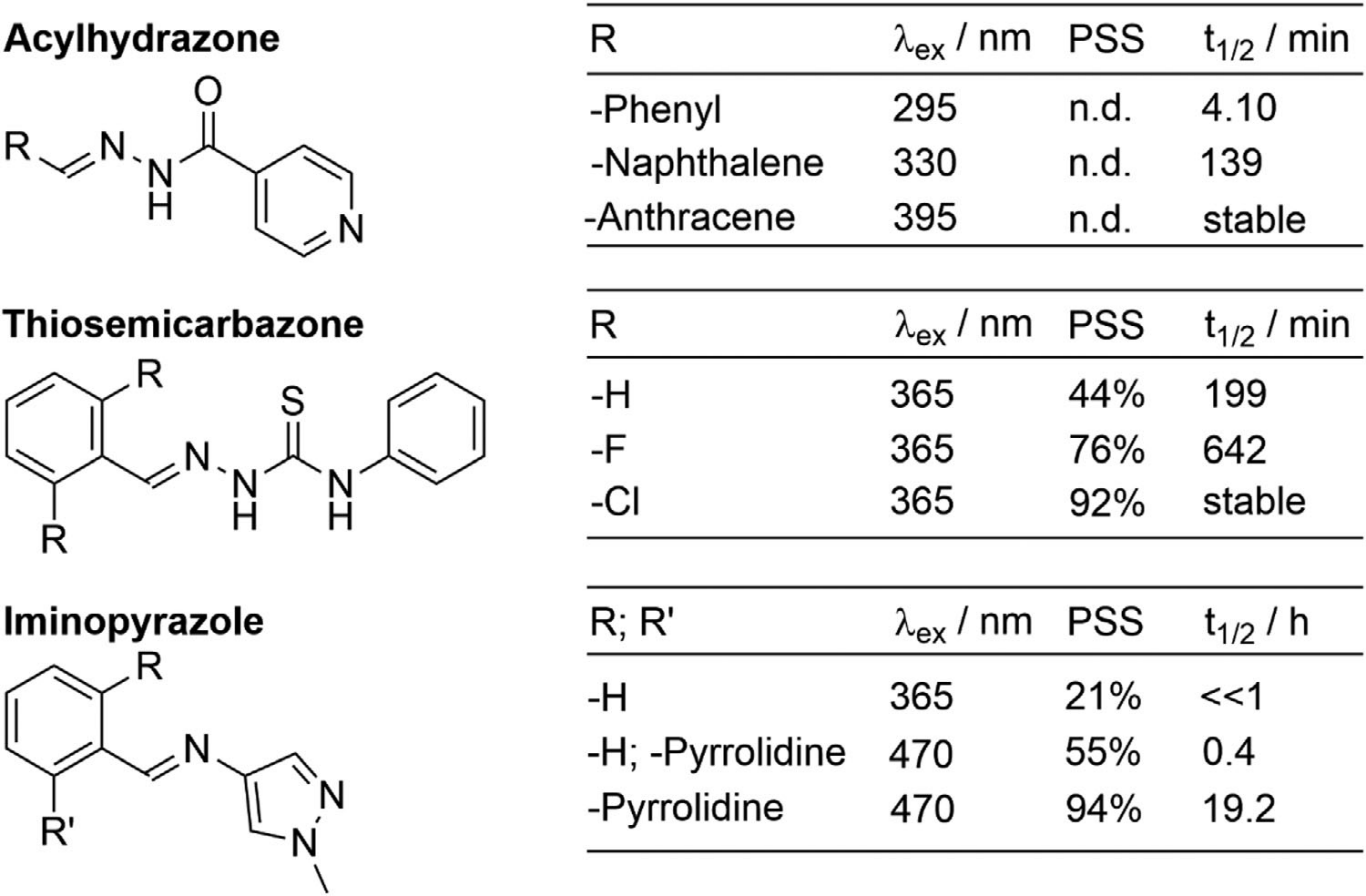
Molecular structures and optical properties of photoswitches involving C = N bond isomerization. λ_e__x_ corresponds to the excitation wavelength applied to the most thermodynamically stable isomer; PSS is the photostationary state composition after irradiation of this isomer; t_1/2_ represents the thermal half‐life of the less stable isomer.

**Thiosemicarbazone**. The group of Eisenreich recently introduced thiosemicarbazones as a promising class of supramolecular organic photoswitches (Figure 7). These molecules exhibit high PSS and tunable half‐lives ranging from minutes to several hours, particularly when halogens are used as ortho substituents. They also demonstrated a bathochromic shift through the conjugation of multiple aromatic rings and enhanced absorbance band separation when the phenyl ring is substituted with heterocycles. With its metal‐ion binding capability, this library of thiosemicarbazones shows great potential for light‐induced biological applications as it can form Cu(II) or Zn(II) complexes to promote anticancer or antimicrobial effects [65, 66, 67, 68].

**Iminopyrazole**. Iminopyrazoles (Figure 7) also act as organic photoswitches which can push dynamic combinatorial libraries out of equilibrium upon light irradiation [69]. The Greenfield group reported a study of aryliminopyrazoles (AIPs), which showed promising properties in comparison to their nonheteroaryl analogues, such as high PSS (up to 95% *Z*‐isomer), lifetime of ca. 19.2 h at room temperature, or negative photochromism that makes them very interesting for applications requiring deep light penetration [70]. Addition of pyrrolidine substituents at the ortho position of the phenyl group stabilizes the metastable form through noncovalent interactions [71]. Iminobispyrazoles (IBPs) further expand this versatility by offering tunable optical properties by varying the number and position of nitrogen atoms in their aromatic rings, altering conjugation pathways. The bathochromic effect resulting from full conjugation of the two pyrazoles does not require ortho electron‐donating groups to act as efficient photoswitches, unlike simple iminopyrazoles [71]. These properties make iminopyrazoles easily functionable to modulate photoswitching properties, with a dynamic covalent chemistry suitable in pH‐sensitive biological environments.

## 3 Covalent Modification of Nucleic Acids by Photoswitches

The utilization of a chemical system that allows the control and manipulation of nucleic acid structures through the action of light is of great interest for the development of target drugs, since light is noninvasive, bio‐orthogonal, and has a high spatiotemporal resolution of action with a view to possible therapies. Based on this idea, a possible approach for the photochemical control of the structure and functions of nucleic acids would consist of the covalent integration of photoswitches in nucleic acids [8, 72, 73, 74].

### 3.1 Photoswitches Incorporated at a Nucleobase Position

**FIGURE 8:**
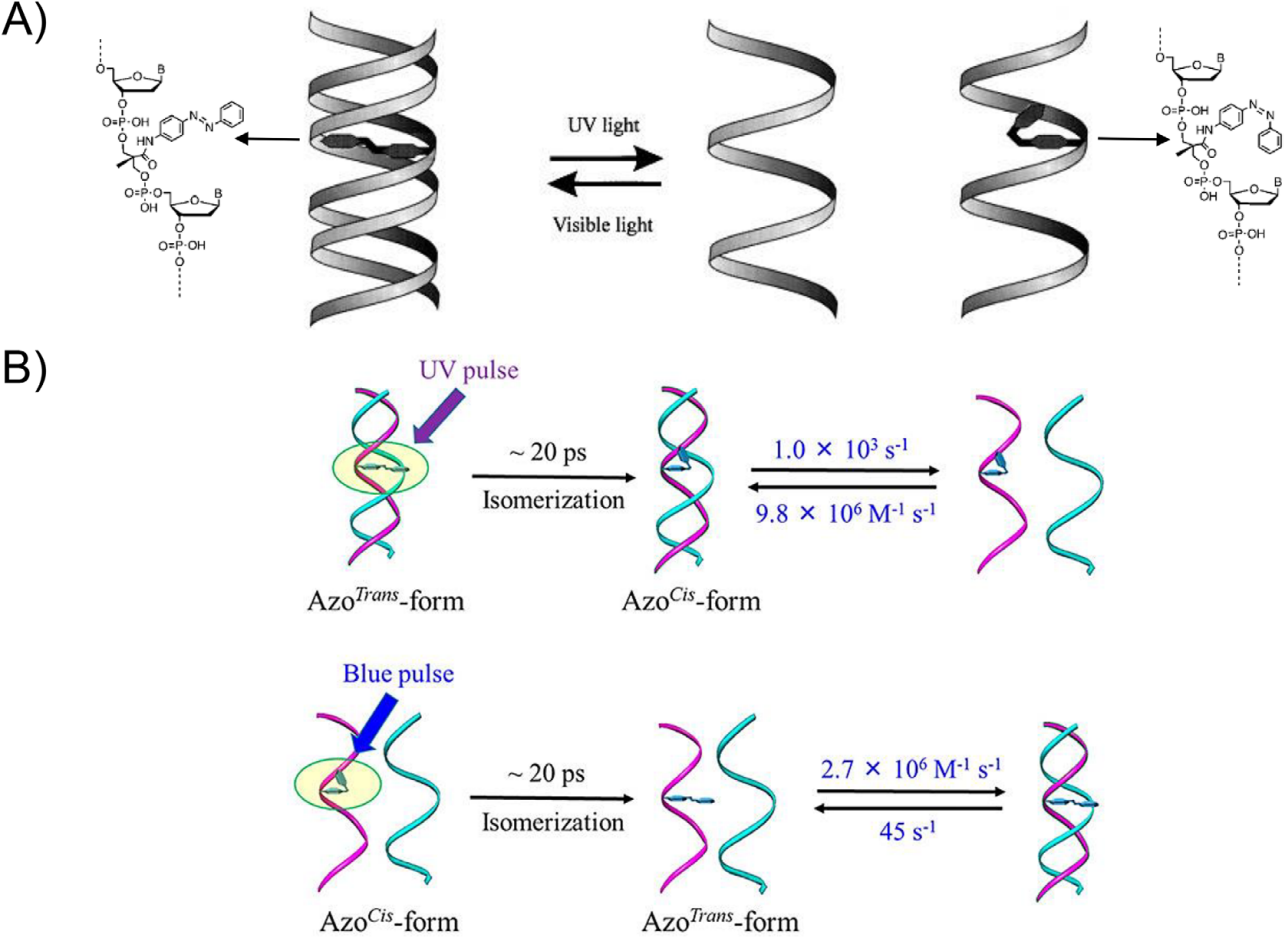
(A) Schematic illustration of the photoregulation of duplex formation by using photoisomerization of azobenzene units, adapted from reference 12. (B) Rate of azobenzene isomerization and rate of dissociation of double‐stranded DNA to single‐stranded DNA triggered by *trans* to *cis* isomerization, adapted from reference [76].

The covalent modification of DNA by a photoswitch was reported by Asanuma et *al*. in 2001 [12]. They introduced a photoresponsive azobenzene group into oligonucleotides in view of controlling the formation/dissociation of DNA duplexes (Figure 8A). In such a system, the melting temperature (*T_m_*), characterizing the dehybridization of DNA duplex into separated strands, changes significantly by induction of *trans* (24.8°C)—*cis* (15.9°C) isomerization, regardless of the position of the azobenzene unit in the oligonucleotide; the change in *ΔT_m_*, induced by the *trans*‐*cis* isomerization, is around 9°C. This indicated that the *trans* isomer of azobenzene stabilized the duplexes by intercalation, whereas the *cis* isomer destabilized the duplex. This showed that photoregulatory oligonucleotides can be successfully used as modulators of DNA elongation by T7 polymerase [75]. This means that photoinduced *cis*‐*trans* isomerization of azobenzene is key in determining whether DNA elongation ends at the desired site or continues to the end of the template DNA.

In 2006, the same group reported on the diffusion dissociation/association method of photoresponsive DNA, in which they showed that although azobenzene isomerization occurs in the order of picoseconds, the dissociation of double‐stranded DNA to single‐stranded DNA triggered by *trans* to *cis* isomerization takes place ∼10^7^ times slower (Figure 8B). In that work, they showed that using the fraction and concentration of azo‐DNA they could calculate the concentration of double‐stranded (dsDNA) and single‐stranded (ssDNA) forms for different temperatures and concentrations, and thus determine dissociation rate and association rate. In addition, the reaction rate of single‐stranded to double‐stranded DNA after *cis*‐to‐*trans* isomerization was determined, as well as the difference in DNA melting temperatures between *trans*‐azobenzene and *cis*‐azobenzene dissociation of the double‐stranded form [76].

More recently, the Ginger group showed that the quantum yield for *trans*‐*cis* photoisomerization of azobenzene decreases in DNA and is sensitive to both local DNA sequence and DNA hybridization state [77]. The photoisomerization quantum yield tends to increase as the *T_m_* of the bound dsDNA decreases. On the other hand, the largest variations in quantum yield are associated with dsDNAs that have a single‐base mismatch right next to the azobenzene site. They measured the rate of disaggregation of nanoparticle conjugates cross‐linked by the same target DNA. This can be useful for the design of systems to photomodulate gene regulation and drug delivery processes.

Finally, in 2016, Santer and colleagues synthesized a photosensitive peptidomimetic molecule (Azo‐PM) for reversible DNA compaction [78]. This research shows how the addition of Azo‐PM to DNA above certain concentrations results in DNA compaction, which was studied by dynamic light scattering (DLS) and atomic force microscopy (AFM). Based on the concentration of Azo‐PM used, they identified three distinct phases: (1) spiral, noncompacted state, (2) reversible compacted state, and (3) irreversible compacted state.

### 3.2 Photoswitches as DNA End‐Groups

**FIGURE 9:**
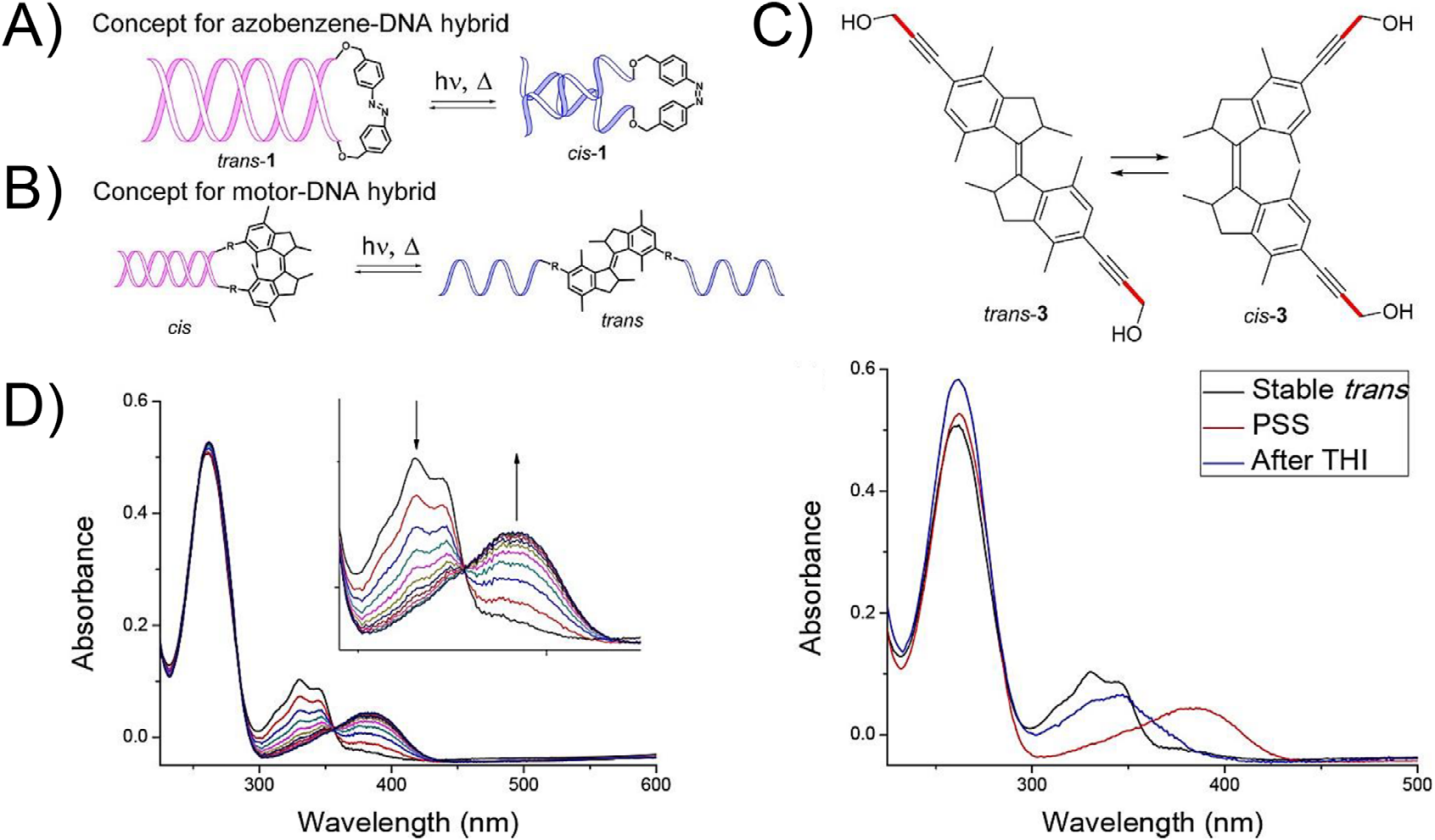
Schematic overview of photoswitchable DNA hairpins. (A) Design by Sugimoto and co‐workers based on photo‐switchable linker 1 [79]. (B) Concept for a linker based on first‐generation molecular motors. A full conversion from double‐stranded to single‐stranded is an unlikely overestimation for both designs, but serves to illustrate the general concept of destabilization through contraction (a) or expansion (b) of the linker [80]. (c) Structure motor linker 3 [80]. (d) UV–vis spectra of analysis of the photochemical isomerization of stable 8T‐*trans*‐3‐8A. Adapted from reference 80.

In contrast to the use of azobenzenes as a photoswitch for DNA hairpin linker (Figure 9A) [79], Feringa and coworkers showed that a first‐generation molecular motor (i.e., 8T‐*trans*‐3‐8A) can function as a photosensitive bridgehead for an 8‐base‐pair DNA hairpin, complementary to their experiments (Figure 9B–D) [80]. Molecular dynamics calculations showed that there were more interactions between the hairpin and the photosensitive bridgehead. In addition, they showed that the isomerization process was highly efficient, and the bistable switching mode was a real advance over *cis*‐*trans* azobenzenes limits possible applications. This work demonstrated that a molecular motor can be used to control the secondary structure of DNA under physiological conditions, demonstrating the ability to regulate a key biological process such as DNA hybridization.

**FIGURE 10:**
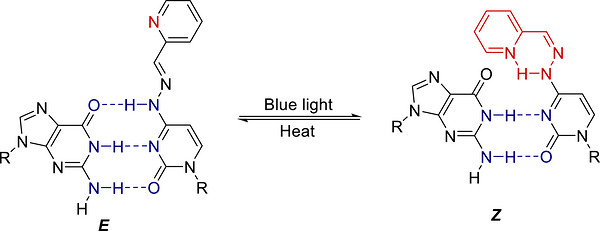
Schematic illustration of light control of the G:C base pair of the hydrazone‐DNA photoswitch [64].

Sheng's group developed a new cytidine‐phosphoramidite family modified with hydrazone and incorporated into oligodeoxynucleotides to construct photosensitive DNA strands by solid‐phase synthesis, Figure 10 [64]. Blue light irradiation‐activated *E*‐isomerization at 450 nm wavelength was investigated and confirmed by proton NMR and HPLC. They reported that the light‐activated *Z*‐form isomer of this hydrazone‐cytidine, with a six‐membered intramolecular hydrogen bond, inhibited DNA synthesis. Furthermore, another result of their research was that cytidine modified with hydrazone in the template could cause dATP disincorporation together with dGTP on the growing DNA strand with similar selectivity, thus manifesting a possible G to A mutation.

## 4 Noncovalent Interactions Between Nucleic Acids and Photoactive Molecules

Instead of introducing photoswitches within the covalent framework of nucleic acids, which is often a nontrivial synthetic task, one can also design photoswitchable ligands which reversibly interact with nucleic acids through noncovalent interactions.

### 4.1 Photocontrol of Nucleic Acid Binding

**FIGURE 11:**
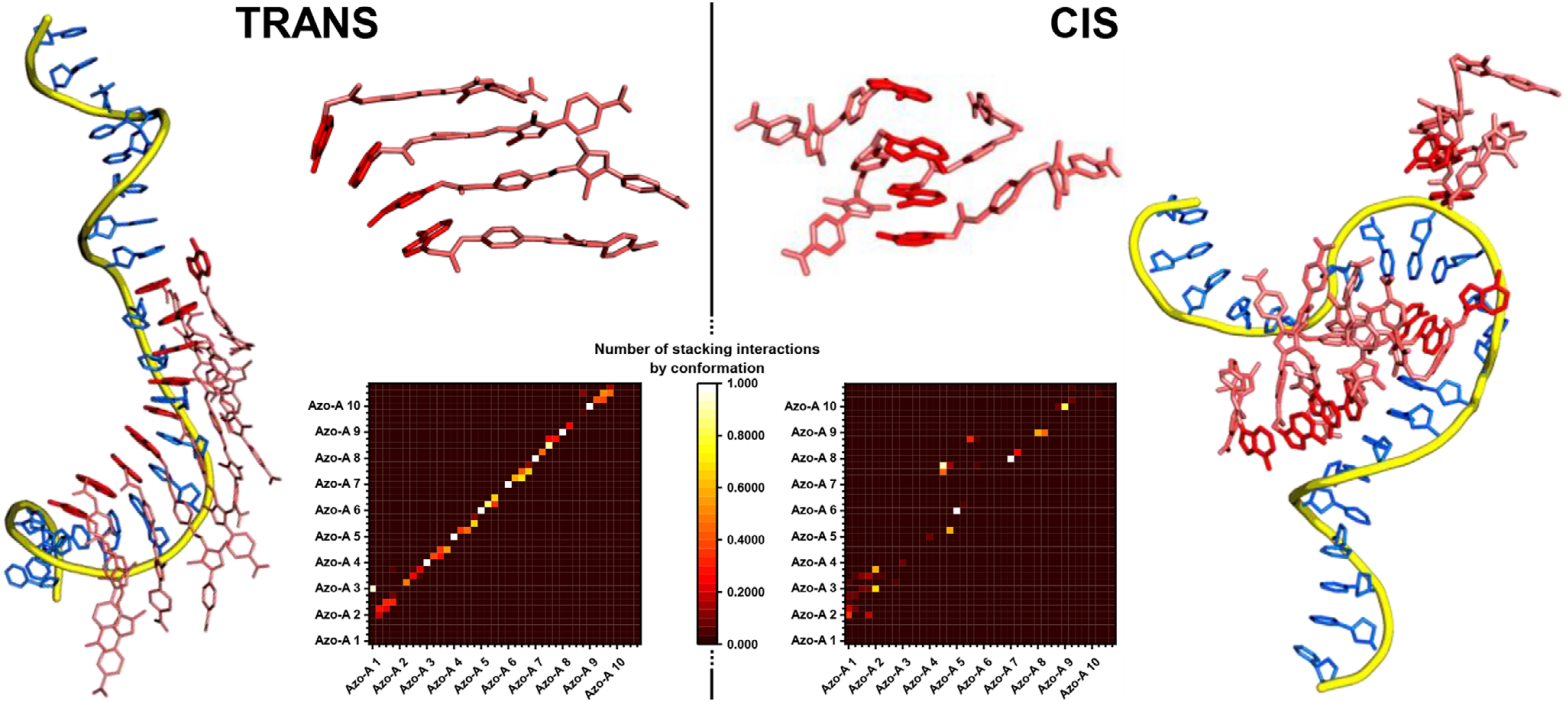
MD snapshots illustrating the Azo‐A/dT_20_ supramolecular complex after 1.25 µs (1 µs without restraints), with the azo compound in the *trans* (left) or *cis* (right) configuration. A zoom on 4 molecules (top center) shows the organization of the stacking for each isomer. The DNA backbone is shown as a yellow tube, its nucleobases are colored in blue, the Azo‐A molecules are represented as pink sticks, and their nucleobases are colored in red. Heatmaps of the stacking interactions between the molecules, calculated on the last 500 ns of the simulation, are shown (bottom center). 40 residues are represented on the x and y axes, one for each aromatic cycle (4 per azo molecule, the molecules being numbered from Azo‐A 1 to Azo‐A 10). At the crossing of a pair of two residues is located a colored square, whose color indicates the number of stacking interactions detected between this pair. The *trans* isomer shows a “diagonal pattern”, meaning that the molecules are stacked consecutively (Azo‐A 1 interacts with Azo‐A 2, Azo‐A 2 interacts with Azo‐A 3, and so on). The *cis* isomer shows more dispersed interactions, with less organization [33].

Nucleic acids, such as DNA and RNA, have the ability to serve as templates for various supramolecular assembly processes, including groove binding, intercalation, and/or base pairing [81, 82]. In the latter, nucleobases act as recognition sites for the binding of synthetic ligands through a selective pattern of hydrogen bonds whose strength is reinforced by π‐π stacking interactions with neighboring nucleobases. Thus, single‐stranded nucleic acids are the simplest platform for studying such supramolecular systems templated by nucleic acids through base pairing, where the nucleic acid‐bound assemblies will form a structure‐ and sequence‐organized array from which emerging function can arise. Interestingly, changing the conformation of such nucleic acid binders shall enable to modulate and control the associated functions. Along this line, the groups of de la Escosura and Surin reported the design of a new type of arylazopyrazole‐derived molecular photoswitches containing a nucleobase (adenine or thymine) in their structure, which interact by base pairing with complementary single‐stranded DNA (dTn and dAn) of various lengths (n = 20 or 40) [33]. To characterize the templated self‐assembly of photoswitches along single‐stranded DNA and the response to light, UV‐Vis absorption spectroscopy and Circular Dichroism (CD) measurements were carried out, varying the solution conditions (ionic strength, [NaCl] = 1 or 5 M) as well as the DNA template used (dT_n_ and dA_n_, n = 20 or 40). An induced CD (ICD) signal was observed, whose sign was dependent upon the nature of the ssDNA template, thereby revealing the selectivity in the chiral organization of the resulting assemblies. Irradiation with UV light (365 nm) induced a chiroptical response, precisely a disappearance of the ICD signal, which was partially restored upon blue light 465 nm) irradiation, thus showing the reversible desorption of the ligand for the ssDNA template. Molecular dynamics simulations support the experimental observations and shed light on the interaction between the DNA strands and the molecular switch in both *trans* and *cis* forms, Figure 11 [33].

**FIGURE 12:**
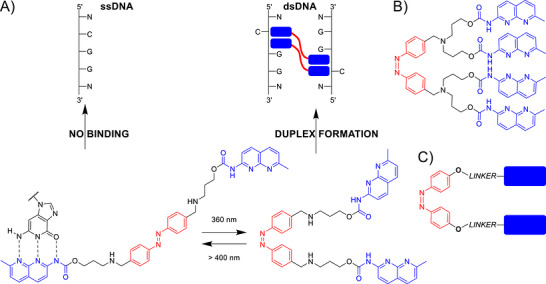
Molecular structures of photoswitchable ligands of (A) DNA, and (B) RNA mismatch duplexes, along with the schematic representation of the optimized photoswitch (C).

Double‐stranded nucleic acids are more complex but form the basis of functional nucleic acids in cells. As early as 2005, light‐activated intercalators of DNA/RNA were described, however then only operating irreversibly through a photoinduced dehydrocyclization [83]. Nakatani and co‐workers reported an early finding on photoswitchable molecular glue for the recognition of DNA [84]. The system is based on an azo‐type photoswitch appended on both sides with a naphthyridine carbamate, which binds to GG‐mismatches (Figure 12A). The results show that only the photoswitched *cis* isomer bind to an 11‐mer mismatch DNA duplex, thereby promoting its hybridization. The reversible photoswitch between the single and double‐stranded DNA which can be achieved using this controllable ligand, was further evidenced by surface plasmon resonance. Beyond these simple nucleobase recognition elements, it is also possible to attach more complex peptide sequences and thereby build photoresponsive DNA‐binding systems [85].

This approach was extended to RNA binding, and the same group showed the stabilization of double‐stranded RNA containing a 5’‐WGG‐3’/5’‐WGG‐3’ (W = U or A) mismatch by the *cis* form of an optimized azo‐based ligand (Figure 12B) [86], achieving a proof‐of‐concept in the photocontrol of the RNA‐cleaving activity of a hammerhead ribozyme in vitro and in cells. More recently, the Dohno group demonstrated that the back‐and‐forth photoswitching under UV and visible light dissociates RNA aggregates of the UGGAA repeat RNA foci, which are involved in neuromuscular diseases [87].

A collaborative work between the Feringa and Dohno groups on the rational design and computationally‐guided design of optimal photoswitches led to the identification of a new set of photoswitchable mismatch‐binding ligands with enhanced band separation of the *E* and *Z* isomers, enabling the photoswitching to operate at 365 and 445 nm, simply by using 4,4’‐di‐alkoxy‐substituted azobenzenes (Figure 12C) [88].

**FIGURE 13:**
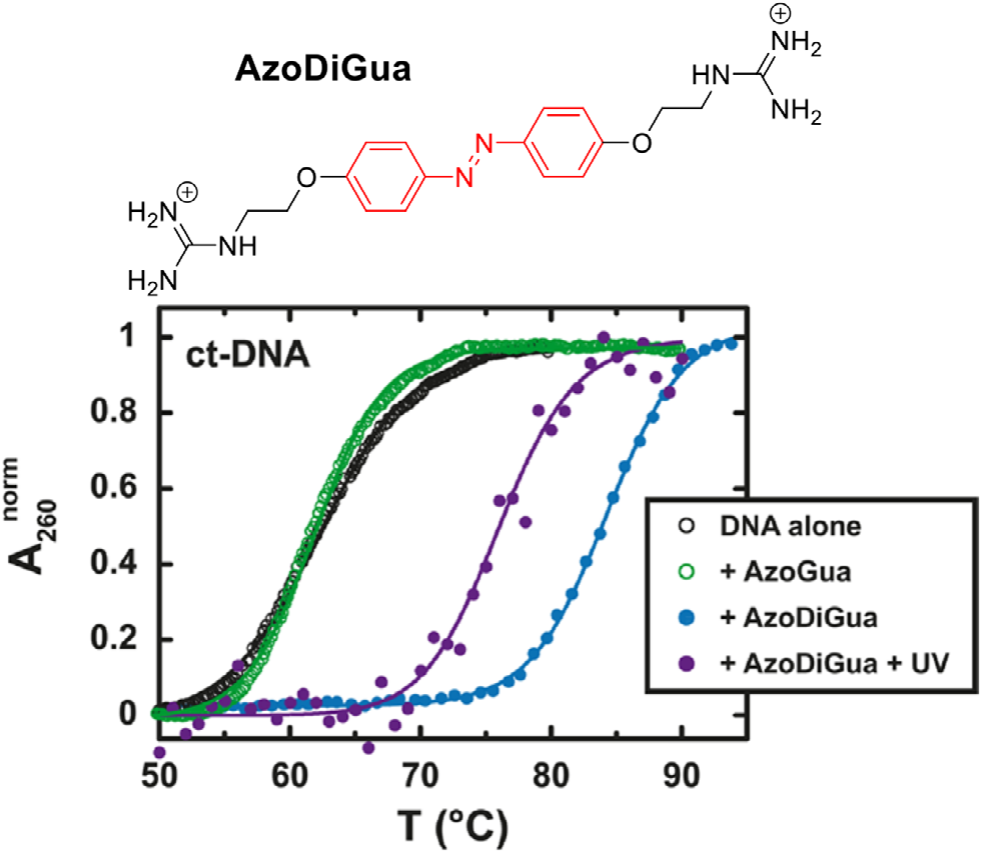
Molecular structure of sequence‐independent photosensitive nucleic acid binder AzoDiGua for the photocontrol of DNA melting, measured by changes in absorption at 260 nm. Figure adapted with permission from reference [89].

Exploiting salt‐bridge interactions with the polyanionic backbone of nucleic acids, the Baigl group studied bisguanidylated azo‐based photoswitchable ligand AzoDiGua and showed the prominent effect on the photoswitching process on DNA melting: + 18°C in melting temperature (*T_m_*) upon photoswitching from *cis* to *trans* isomers because the former does not bind strongly, whereas the latter intercalate in, and therefore stabilizes, the DNA double helix (Figure 13) [89]. Since the interaction with DNA is mainly electrostatic (ionic interactions), the process works equally well for short DNA (10 base pairs) up to long genomic DNA (calf‐thymus, > 10 kbp). Matczyszyn and co‐workers have subsequently reported an improved design which integrates ortho‐fluoroazobenzene which can now be photoswitched with visible light (405 nm from *cis* to *trans*, and 515 nm from *trans* to *cis*) [90].

**FIGURE 14:**
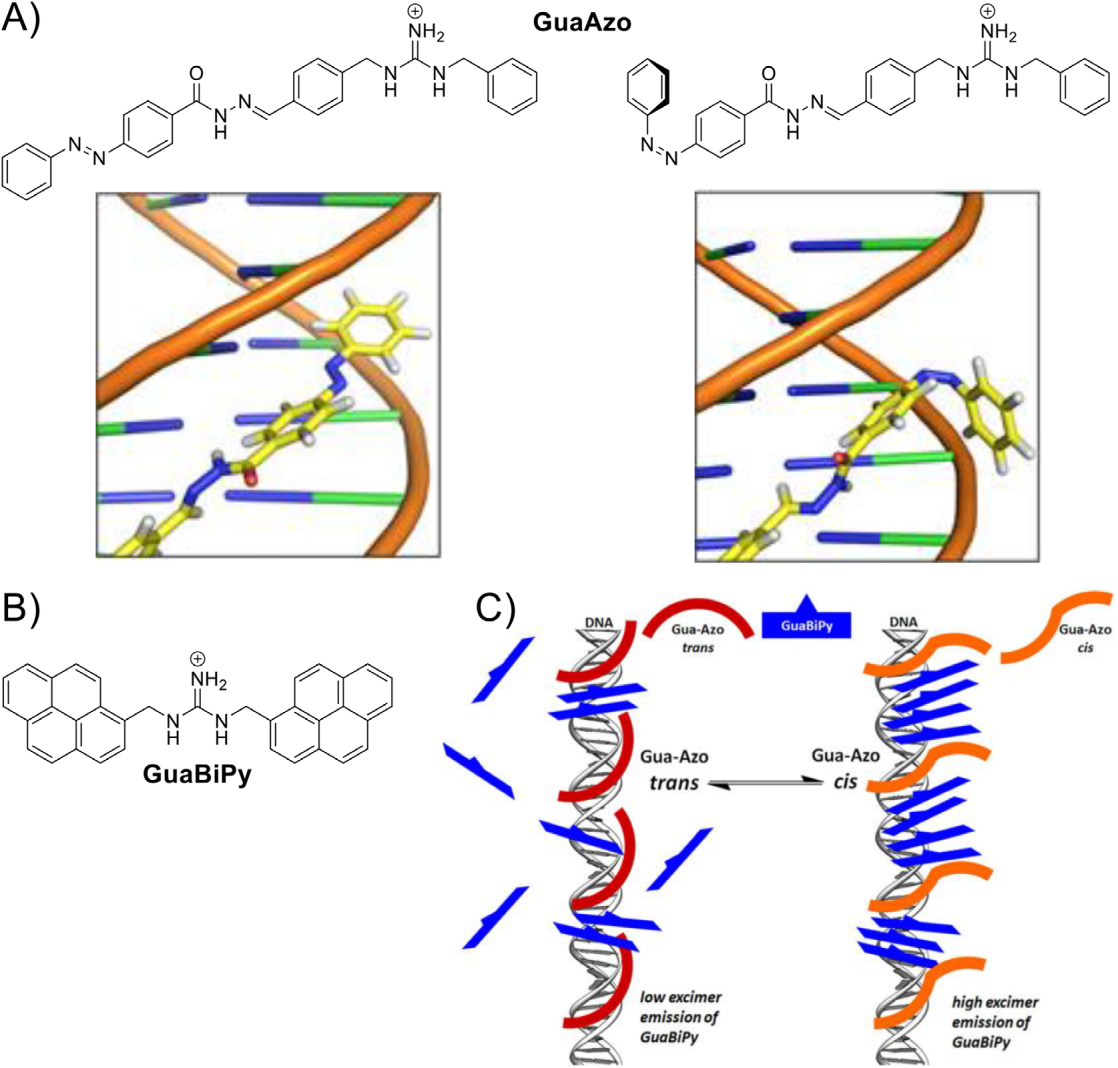
Representation obtained by molecular modelling of the photomodulation of dsDNA binding by the two *trans* and *cis* isomers of GuaAzo ligand (A); molecular structure of GuaBiPy (B); and schematic representation of the photomodulation of a chemical system where the two ligands compete for dsDNA and this light‐dependent competition outcome results in a controlled fluorescence output originating from the excimer assembly of GuaBiPy (C). Adapted with permission from reference [92].

The Surin and Ulrich groups studied small‐aromatics bearing guanidinium groups as phosphate‐binding DNA ligand and showed the importance of π interactions in stabilizing the resulting self‐assemblies with ssDNA [91]. Now incorporating a photoresponsive azobenzene moiety in the design of the ligand, it was found that desorption from ssDNA occurs when photoswitching from *trans* to *cis* isomers. This is best explained, and confirmed by molecular modelling studies, by a modulation of the planarity of the system which alters π interactions (Figure 14A). Now moving to dsDNA, it was found that, when the azobenzene is in the *trans* isomer, this ligand binds in the minor groove of a double‐stranded DNA, whereas it partially desorbs from the minor groove upon *trans–cis* isomerization with light (Figure 14B). The differences in binding geometry, stability, and dynamics of the DNA/ligand complexes for the two isomers have been studied. The properties of this ligand were subsequently used to photomodulate a complex system where two ligands (GuaAzo and GuaBiPy) compete for the same DNA template. With the *trans* isomer of GuaAzo, the two ligands compete equally well, and the mixed binding results in a weak excimer signal. In contrast, this excimer signal sees a twofold increase after photoisomerization, which is best explained by the partial desorption of the *cis* isomer of GuaAzo which leaves GuaBiPy forms more homogeneous stacks on the DNA template. This study demonstrates that well‐designed photoresponsive DNA binders can be used to modulate multicomponent supramolecular DNA assemblies, Figure 14C [92].

Beyond the photocontrolled association with single‐ and double‐stranded DNA and RNA, the use of switchable ligands also bears a strong interest for the recognition of nucleic acid structures of higher complexity which dynamic formation is involved in the control of gene expression. The use of photoswitchable ligands can thus be of interest for modulating the downstream expression of RNA. The group of Galan reported on a stilbene‐based photoswitch for the recognition of G‐quadruplexes DNA [93]. Depending on its isomerization, the ligand was found to stabilize or not the G‐quadruplex. Very recently, the group of Balasubramanian reported a cyclic azo (Diazocine) G‐quadruplex ligand which reversible photoswitching results in a spatiotemporal control of the expression of a set of G4‐dependent genes [94]. Small‐molecule groove binders also form a set of selective RNA‐binding ligands [95, 96], recognizing specific folded secondary structures of nucleic acids. The group of Vázquez reported a small molecule that enables conditional real‐time control at mRNA and protein levels [97]. The RNA binding compound features an ortho‐fluoro azobenzene which binding to the ssRNA binding site 2A in the exon 7 of the SMN2 pre‐mRNA. Photocontrol of RNA splicing was then shown in cells.

### 4.2 Photocontrol of DNA Folding and Condensation

**FIGURE 15:**
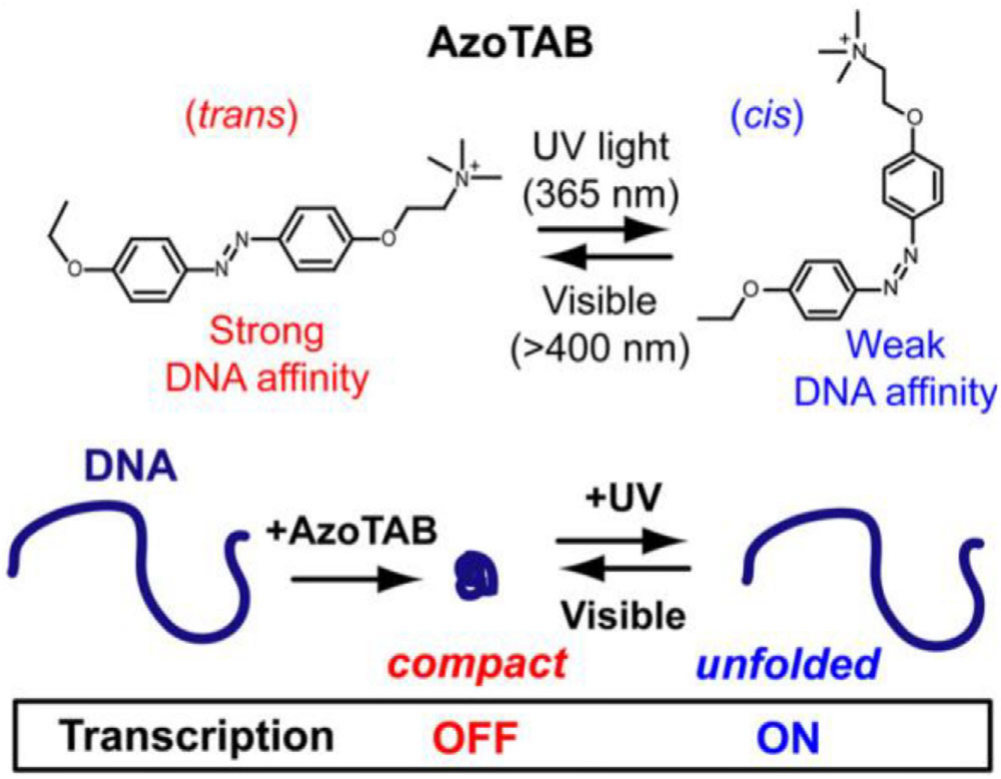
Molecular structure of sequence‐independent photosensitive nucleic acid binders for the photoregulation of transcription. Reproduced with permission from reference [98].

The group of Baigl extensively studied cationic surfactants as sequence‐independent nucleic acid binders and reported, by introducing a photosensitive azo moiety with the compound AzoTAB, on the photocontrol of transcription and translation processes with binders that are active in the dark and inactivated upon UV light irradiation (Figure 15) [98]. The design rests on an azo photoswitch, splitting a permanent cationic head from a short lipophilic tail, which isomerization affects nucleic acid binding and condensation [99]. As a consequence, accessibility to the enzymatic transcription machinery is altered by the photoswitching of the ligand.

**FIGURE 16:**
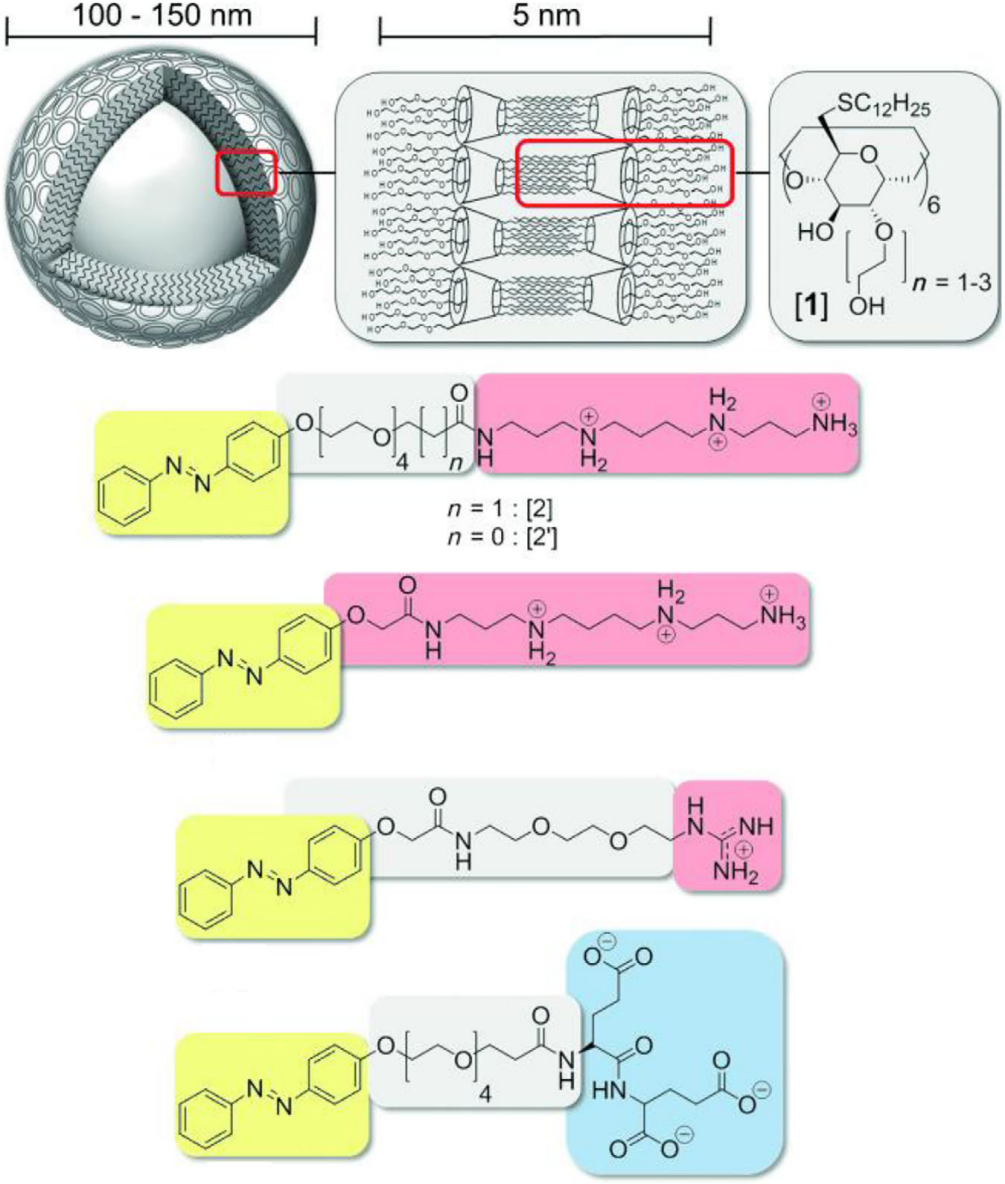
Molecular structure of photoswitchable cationic guests which, upon combination with amphiphilic cyclodextrin hosts, form cationic vesicles able to complex DNA and release DNA after light irradiation. Reproduced with permission from reference [100].

The ability to control the complexation and condensation of nucleic acids opens perspectives for application in DNA or RNA gene therapies since these processes are underlying the formation of nanoparticles, which can traffic in biological fluids and pass biological barriers to reach their targets. In this line, the Ravoo group reported on vesicles made of cyclodextrins hosting azo‐based photoswitchable cationic amphiphiles able to reversibly complex DNA (Figure 16) [100, 101]. There, the photoswitching of the azobenzene triggers dissociation of the host‐guest complex with cyclodextrin, which, in turn, leads to the controlled release of DNA.

In contrast to caged RNAs [102] or RNA delivery vectors which use photolabile groups for the irreversible release of nucleic acids [103], the use of reversible photoswitches suppress the concomitant release of an accumulating small‐molecule side product and offer temporal and spatial control [104] as the delivery can be stopped with light irradiation after some time, and possibly also in areas where RNA delivery is not desired. Such an approach will contribute to making smart addressable vectors for RNA delivery [105, 106]. For instance, developments in the design and application of photocontrolled lipids [107] shall undoubtedly translate into smart lipid nanoparticles for RNA delivery with improved efficacy.

### 4.3 Photocontrol of DNA‐Based Hydrogels and Higher‐Order Nanostructures

DNA hydrogels are attractive materials as they combine biocompatibility and recognition properties, while their mechanical properties can be tuned through a proper supramolecular design. Besides, they hold great promise to impart stimuli‐responsivness and adaptive behavior [108].

**FIGURE 17:**
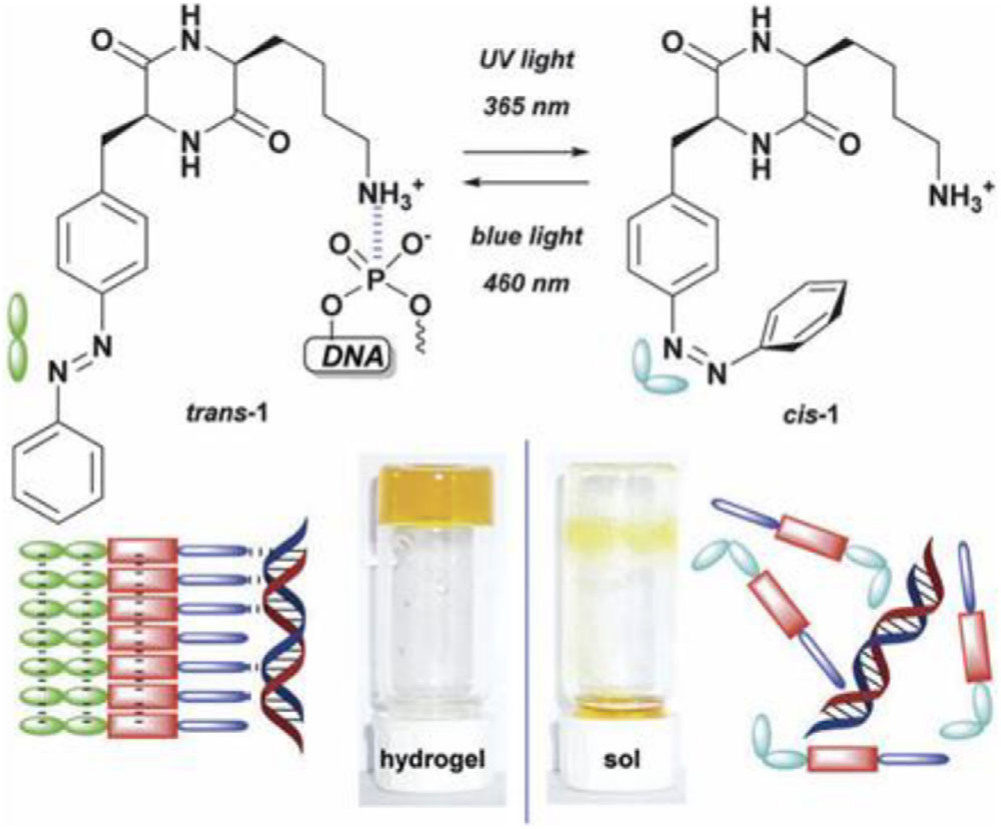
Supramolecular hydrogel forms from a cyclic dipeptide‐based gelator in aqueous solution. Reversible light‐controlled sol‐gel transition [109].

For example, Pianowski and co‐workers developed light‐responsive supramolecular hydrogels that are able to encapsulate therapeutically‐relevant molecules and release them in response to UV light irradiation. Supramolecular hydrogels are based on cyclic dipeptide‐based gelators bearing a photoswitchable azobenzene group (Figure 17). In solution, the gelator molecules in *trans* configuration self‐assembled through the π‐stacking of aromatic azobenzene moieties and the hydrogen bonds of the diketopiperazine rings, forming a fibrous network as confirmed by electron microscopy. Upon UV irradiation, the gels rapidly turned into a liquid. This phenomenon is due to the photoisomerization of azobenzene from the *trans* to *cis* configuration, which disrupts interactions leading to fiber disassembly and gel degradation. Conversely, blue light irradiation for 30 min induced the reverse isomerization, increasing the population of *trans* isomers and ultimately regenerating the fibrous gel structures. It should be noted that the gelation process requires several hours to yield a hydrogel with sufficient mechanical stability. This strategy was successfully employed to achieve the light‐induced delivery of oligonucleotides and the anticancer drug doxorubicin, highlighting its potential as a platform for the development of stimuli‐responsive drug delivery systems toward therapeutic materials [109]. More recently, Pianowski's group engineered hydrogels based on this gelator that release previously encapsulated drugs under visible light irradiation by substituting the azobenzene unit with halogen groups [110].

Recently, Feringa's group reported the design of a DNA‐based hybrid supramolecular hydrogel whose structure and properties can be reversibly modulated by light and thermal stimuli [111]. The system is built upon self‐assembly of a dithienylethene (DTE) photoswitch bearing two adenine units with a 20‐thymine single‐stranded oligonucleotide template. In self‐assembly, the photoresponsive molecule enables reversible switching between distinct functional states. Circular dichroism and microscopy analyses revealed that supramolecular assemblies are stabilized via hydrogen bonding interactions between dithienylethene photoswitches and the DNA single‐strand, which forms aggregates as kinetic product. The aggregates can evolve to an equilibrium state consisting of a homogeneous network of twisted ribbons, ultimately forming tightly packed fiber bundles that give rise to a self‐healing supramolecular hydrogel. At the macroscopic level, gels exhibit unusual shape‐resilient behavior, retaining the shape of the vessel in which it was formed. Interestingly, exposure to UV light induces localized shrinkage within the gel, enabling a precise light‐controlled modulation of its original shape. Subsequent irradiation of visible light combined with a heating–cooling cycle allowed the recovery of the kinetic product. This strategy provides a dynamic and reversible platform for controlling supramolecular architectures and their properties at the microscopic and macroscopic scale.

Additionally, the emerging field of DNA nanotechnology strips this molecule of any preconceived biological function and exploits its simple code to generate addressable nanostructures in 1, 2, and 3D. Higher‐order structures, i.e., organized DNA nanostructures beyond the double helix, have been used to arrange proteins, nanoparticles, transition metals, and other functional components with deliberately designed patterns. They can also serve as nanowire templates, help to determine protein structure, and provide new platforms for genomic applications. The field of DNA nanotechnology is growing in several directions, and promises to have a major impact on materials science and biology [112].

For example, Sugiyama and co‐workers designed a DNA nanocapsule with a light‐sensitive open/close mechanism based on azobenzene. These researchers exploited these nanocapsules to effectively trap gold nanoparticles via DNA hybridization and released through light irradiation and strand displacement [113].

This group also reported on the design of photosensitive DNA origami nanostructures capable of assembling into programmed, multi‐oriented patterns. They used hexagonal DNA origami units decorated with azobenzene‐modified oligonucleotides, which act as photoswitching molecules. The process of assembly/disassembly between hexagonal DNA units can be reversibly controlled by the wavelength‐dependent *trans*‐*cis* photoisomerization of azo groups. The orientation of hexagonal DNA units relative to each other within self‐assembled nanostructures can be finely controlled by modifying the number and the position of photosensitive oligonucleotides, allowing for a variety of patterns such as linear or ring arrangements in 2D [114]. Later, the same group used arylazopyrazole as a photoswitching molecule due to its superior photophysical properties compared to azobenzene moieties. They demonstrated that azobenzenes and arylazopyrazole photoswitches could be used together to independently control the self‐assembly process, opening up new possibilities for modulating DNA origami nanostructures [115]. Besides, DNA origami nanostructures have also been used as cargo to encapsulate and then release biologically relevant molecules upon light exposure. For example, Xue Han and co‐workers engineered a DNA origami nanocage by introducing photolabile linkers to entrap a wide range of molecules. This strategy has enabled the successful encapsulation of various proteins and then releasing them in their intact, bioactive state when exposed to light [116].

## 5 Summary and Outlook

Early‐generation photoswitches have been successfully combined with nucleic acids, either covalently—replacing internal nucleobases or placed at end‐groups—or noncovalently through supramolecular recognition. Several proof‐of‐principles have been reported on the photocontrol of DNA/RNA hybridization, DNA condensation, DNA origami, and DNA‐based hydrogel formation, ligand binding, and transcription/translation regulation. We also highlighted here the recent progress in the development of new photoswitches, which now provide an expanded repertoire of molecular structures for fine‐tuning the wavelength selection, the extent (PSS), and the kinetics (t½) of photoswitching for the application pursued. These recent developments bring forward new photoswitches with improved properties, for instance, in terms of efficiency and kinetics of photoswitching, and tunable back‐isomerization. Although only a few studies have started this exploration, more advances are yet to come to reveal the potential of photocontrolled nucleic‐acid assemblies for modulating gene expression and in photopharmacology.

## Conflicts of Interest

The authors declare no conflicts of interest.
